# Sudden death associated with lipoma of the cerebellopontine angle

**DOI:** 10.4322/acr.2021.396

**Published:** 2022-08-26

**Authors:** Stefano Tambuzzi, Guendalina Gentile, Michele Boracchi, Arnaldo Migliorini

**Affiliations:** 1 Università degli Studi di Milano, Dipartimento di Scienze Biomediche per la Salute, Sezione di Medicina Legale e delle Assicurazioni, Milano, Italy; 2 Università degli Studi di Milano, Milano, Italy

**Keywords:** Brain Neoplasm, Autopsy, Forensic pathology

## Abstract

Intracranial lipomas are rare benign tumors considered exceptional when localized in the cerebellopontine angle (CPA), with an incidence of 0.1% of the total number of expansive processes located in this area. We present a case of the sudden death of a 26-year-old young woman in which an unencapsulated neoformation of 0.8 cm was documented at the right cerebellopontine angle and was histologically characterized as intracranial lipoma. The cause of death was then identified as a cardiocirculatory failure secondary to supratentorial (uncal right) herniation caused by the lipoma of the pontocerebellar angle with high-grade diffuse cerebral edema.

## INTRODUCTION

Intracranial lipomas are rare benign lesions with an incidence at autopsy of 0.08%.^1^^,^^2^ Lacking cellular atypia, dysplasia, and other malignancy indices, these lipomas are considered embryonic developmental disorders rather than neoplasms.^3^^,^^4^^-^5 Frequently, intracranial lipomas are localized on the midline above the corpus callosum or near the cerebellum and the IV ventricle. The extra-axial sites, are documented in the Sylvian cistern and the cerebellopontine angle (CPA).^3^^,^^6^ The cerebellopontine angle harbors 8-10% of all intracranial tumors,^7^ including vestibular schwannomas (75-90% of cases).^8^ In this site, the CPA lipoma is considered a striking finding, showing an estimated incidence between 0.05%^5^ and 0.1%.^6^^,^^9^

In this report, we present the case of an unexpected death of a young woman affected by cerebellopontine lipoma.

## CASE REPORT

**Figure 1:**
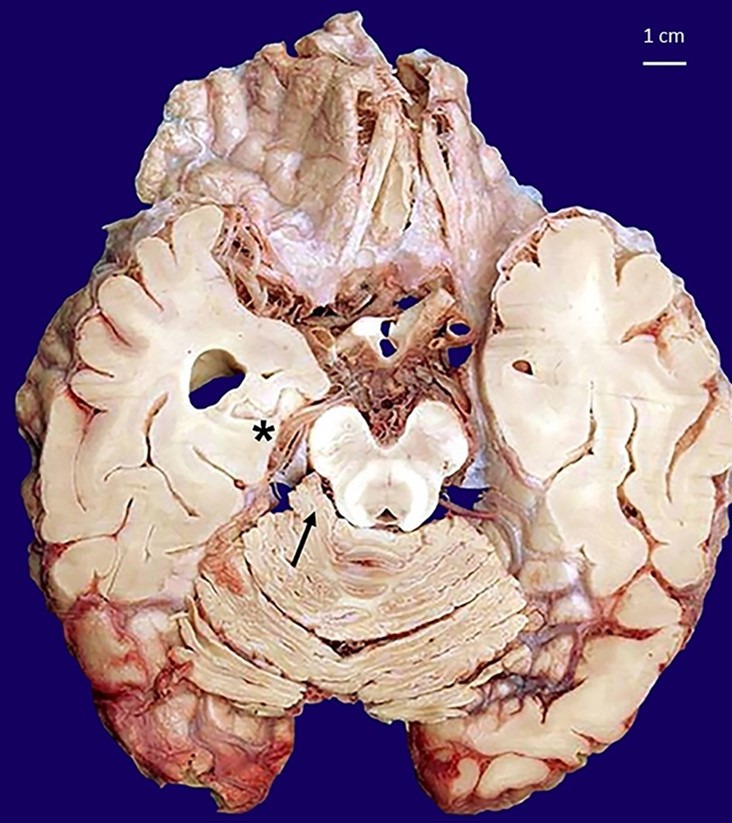
Macroscopic view of a sagittal section of the encephalon fixed in buffered formalin highlights the neoformation in the cerebellopontine angle localization (arrow) and the uncal right herniation (asterisk).

**Figure 2:**
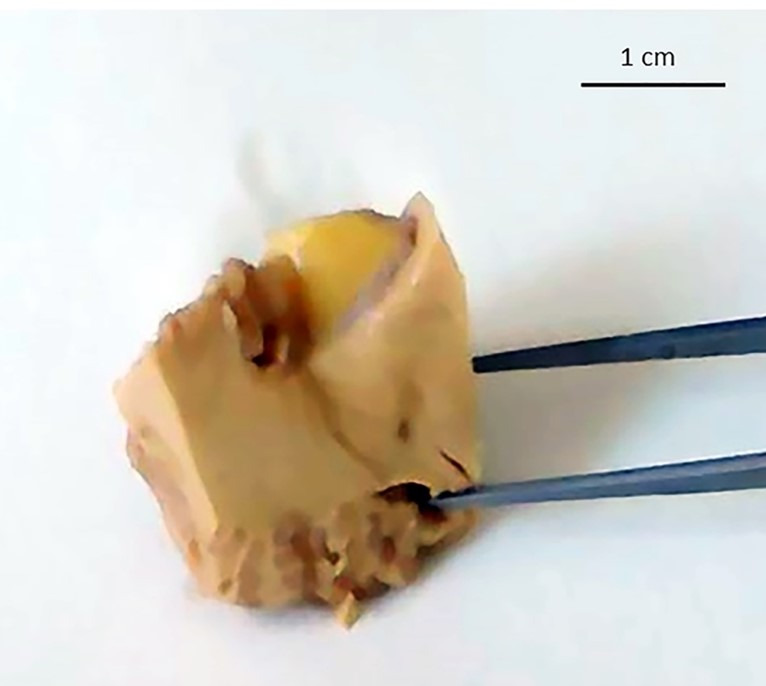
Detailed macroscopic view of a longitudinal section of the yellowish color neoformation in the right pontocerebellar angle.

A 26-year-old girl was found dead in her home. Her family members reported they had seen her alive a few hours before and that she occasionally suffered from migraine, which was never medically investigated and treated with over-the-counter analgesics. They declined alcohol or drug abuse. The autopsy was ordered by the Judicial Authority. At post-mortem examination, the corpse was in good preservation conditions (weight = 52 kg, length = 171 cm). It did not present any sign of traumatic-blunt lesion on external examination. At cadaveric dissection, the brain showed smoothed convolutions and shallower grooves than normally and there was evidence of supratentorial herniation (uncal right) with tonsillar imprint (Figure 1). In the right cerebellopontine subarachnoid area, there was an unencapsulated neoformation in 0.8 cm in diameter with a well-delimited surface of yellowish color and elastic consistency (Figure 2). Nothing else significant emerged from the autopsy examination.

During the autopsy investigation, fragments of viscera and biological fluids were sampled for the usual chemical-toxicological analyses, as well as the brain and heart *in toto* and samples of the lung, liver, kidneys, and spleen for subsequent histopathological study. The latter were conducted using standard post-fixative techniques. The sections obtained were stained using basic H&E and Masson trichrome histochemical staining. Slides were observed with a Leica DMR optical microscope, capturing the most significant images with a Leica DC300F Digital Camera.

**Figure 3:**
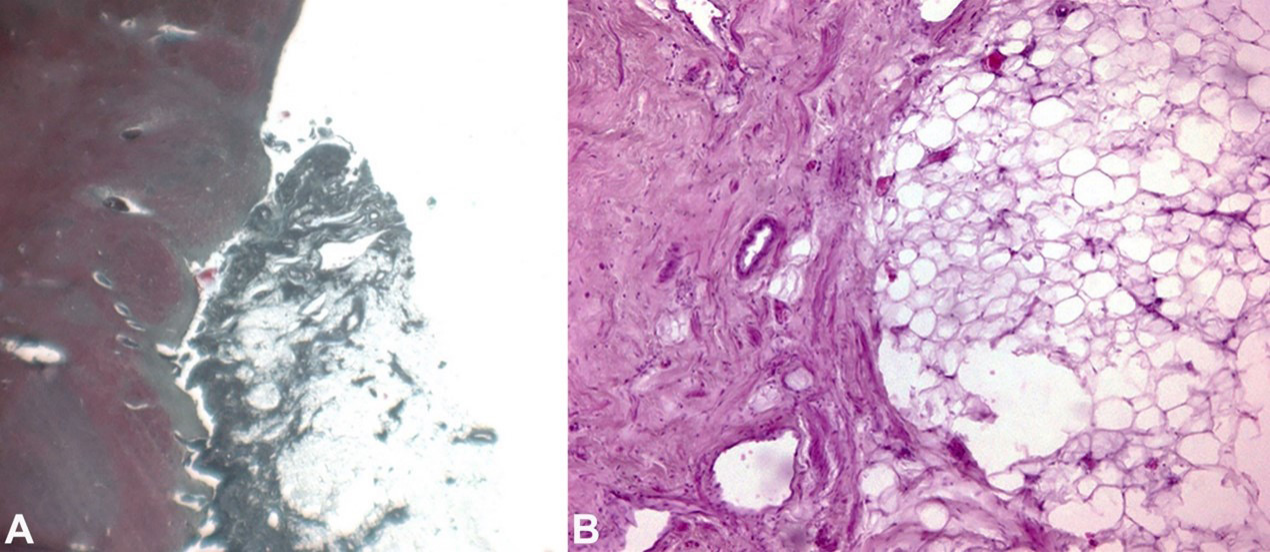
Photomicrograph of the cerebellopontine angle. **A –** adherent lipoma; a neoformation that cannot be separated from the cerebellar tissue with direct contact of the adipocytes with the nervous tissue (Masson trichrome, 50X); **B –** greater magnification of the neoplasm with an external portion consisting of fibrous tissue adhering to the structure (pseudocapsule) rich in small vessels and a central structure with proliferation of mature adipose tissue (H&E, 100X).

Toxicological analyses were negative for determining Blood Alcohol Concentration (BAC), narcotics, and psychotropic substances. On the other hand, histopathological investigations of the viscera and the cerebellopontine neoformation showed a formation composed by mature adipocytes, allowing to define it as an intracranial lipoma (Figure 3).

**Figure 4:**
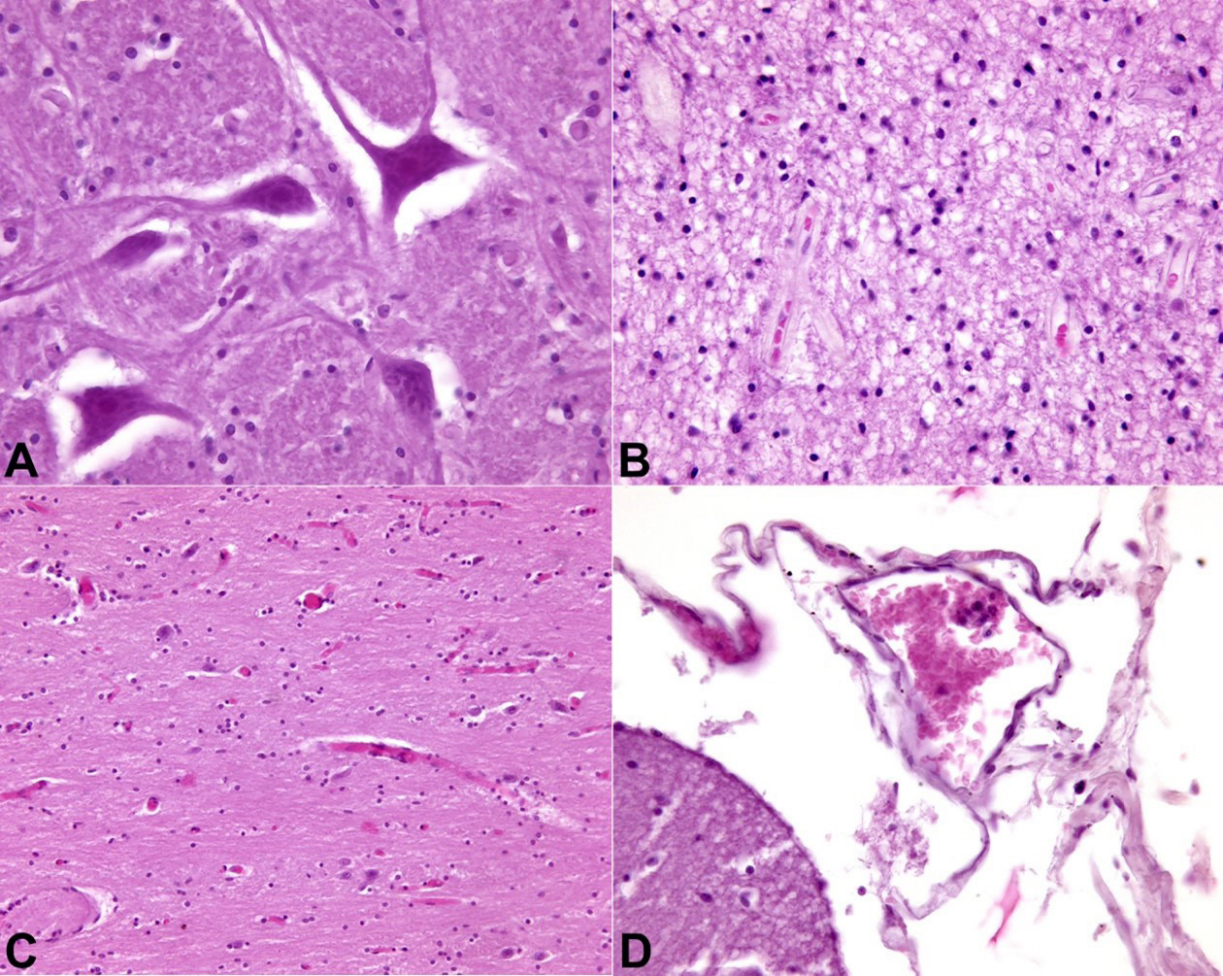
Photomicrograph of the Central nervous system. **A –** olivary body with neuronal eosinophilic degeneration (H&E,400X); **B –** olivary body with tissue spongiosis of the right temporal lobe (H&E, 200X); **C –** right temporal lobe with cortico-subcortical vascular congestion (H&E, 200X); **D –** right parietal lobe with leptomeningeal congestion (H&E, 400X).

No evidence of malignancy was observed. Areas of acute neuronal hypoxic distress in the bulbar site (olive) and diffuse cerebral edema of a severe degree, mainly in the right hemisphere, were also documented (Figure 4).

Histological examinations of all other viscera revealed no pathological findings. In detail, the heart, which was taken entirely, was studied carefully with numerous histological samples, and sudden death from cardiac causes was ruled out.

Therefore, based on the concordance and convergence of the data that emerged at autopsy examination and laboratory testing, we attributed the cause of death of the young woman to acute cardiocirculatory failure secondary to supratentorial herniation (right uncal) from lipoma of the cerebellopontine angle with diffuse cerebral edema of high degree.

## DISCUSSION

Intracranial lipomas (ILs) are benign, slow-growing neoplasms that typically cause progressive neurological symptoms with loss of hearing ability, dizziness, tinnitus, trigeminal neuralgia, and occasionally, involvement of the facial^6^ and trigeminal nerves.^10^^,^^11^^,^^12^^-^13 Macroscopically, although presenting as well-defined masses of adipose tissue incorporating the fibers of the cranial nerves due to the high degree of vascularization, they can be misinterpreted as hamartomas.^6^^,^^14^ Microscopically, ILs consist of mature adipose tissue, large cells rich in lipids, and little or no fibrous stroma, similar to the lipomas found in other body sites. The adipose mass, in its slow expansion, involving nerve bundles, arteries, and venous vessels, realizes tenacious adhesions to the surrounding structures making it difficult to surgically resect it, even partially. Due to the rare incidence of these tumors of the cerebellopontine angle, experience in therapeutic management is limited, and the resection is indicated, by several authors, only in symptomatic cases refractory to targeted medical therapies aimed to alleviate intractable cranial neuropathies or reduce compression on the brain stem.^15^ Most of the CPA lipomas are managed conservatively.^16^ However, when the lesion reaches a considerable size and involves a critical neurovascular structure, causing symptoms, the surgical approach is recommended to pursue the decompression on neural structures.^15^

A case of sudden death of a young woman with a lipoma of the pontocerebellar angle has been illustrated. The deceased presented episodes of migraine, which were never clinically investigated. The post-mortem examination depicted a single pathological cause responsible for the death, which consisted of an expansive intracranial process characterized by mature adipose tissue in the absence of cellular atypia, dysplasia, and other malignant indices on microscopy. The case was considered of particular interest because of the exceptional rarity leading to sudden death, and the lack of alarming symptoms that could eventually indicate a previous diagnostic workup. In detail, it was reported by relatives that the girl had suffered episodes of migraine. However, since no clinical examination had been carried out *intravitam*, the true nature of the headache pathology suffered by the girl remained unclear. In the literature, the correlation between evolving intracranial lipoma (PCA included) and headache is well known and reported on several occasions.^17^^,^^18^ In contrast, no evidence straightforwardly correlates PCA lipoma with migraine. There are two possibilities: either the PCA lipoma caused the headache, or the migraine was more likely independent of the intracranial pathology. Ultimately, therefore, this was a paucisymptomatic or completely asymptomatic case. The young woman did not experience the troubling symptoms that frequently distinguish a PCA, representing a peculiar characteristic of the case. On the contrary, the post-mortem diagnosis was clear, and the macroscopic and histological autopsy findings provided the essential key to demonstrating the cause of death, determined by the lethal central nervous repercussions of a histologically benign entity.
